# The importance of vision care for displaced populations: lessons from Israeli evacuees

**DOI:** 10.1186/s13584-024-00615-x

**Published:** 2024-06-06

**Authors:** Hadas Ben-Eli, Itay Chowers, Ariela Gordon-Shaag

**Affiliations:** 1 Department of Optometry and Vision Science, Hadassah Academic College, 37 Neviim St. Jerusalem, Jerusalem, Israel; 2 Department of Ophthalmology Hadassah, Hebrew University Medical Center, POB 12000, Jerusalem, 91120 Israel

**Keywords:** Vision Care, Humanitarian intervention, Evacuees, Emergency Ophthalmology, Crisis Response

## Abstract

**Background:**

This study explores vision care priorities and coping mechanisms for Israeli evacuees and following the October 7th, 2023, attack by Hamas, which displaced 150,000 individuals, with about 15,000 being evacuated to the Dead Sea area. Faced with minimal health care infrastructure in the Dead Sea area and often lacking personal belongings, including eyeglasses and ocular medicine, these evacuees confronted significant vision care challenges. This context sets the stage for investigating the emergency vision care needs and solutions for populations affected by conflict and displacement.

**Methods:**

In response to this crisis, a consortium led by Hadassah Academic College’s Department of Optometry and the Dept. of Ophthalmology at Hadassah Medical Center established ophthalmic clinics in the Dead Sea region. These clinics offered comprehensive vision care services, including refractive and vision examinations, ophthalmological assessments, ocular imaging, and provision of free glasses. The setup included multiple stations for different vision tests, staffed by an interdisciplinary team of professionals. The study analyzes the effectiveness of these clinics, patient flow challenges, and the psychological impact of vision care in a crisis setting.

**Results:**

Approximately 800 evacuees received examinations, with around 700 pairs of glasses distributed. Notable cases included emergency referrals for serious conditions and instances where glasses served as psychological support. The operation highlighted the necessity of vision care during crises and its potential psychological and social implications. The clinics successfully provided immediate vision care, but challenges in patient flow and insufficient electronic medical record integration were noted. The experience underscores the importance of prepared eye care interventions in crises. Recommendations for health policy decision-makers include establishing a national emergency vision care network, developing standardized treatment protocols, training local health workers, and raising public awareness about eye health in emergencies.

**Conclusions:**

The consortium’s effort in providing urgent vision care to evacuees from the Hamas attack on Israel demonstrates the critical role of rapid, organized eye care in crisis situations. Vision care, along with hearing and mobility, is often overlooked during evacuations but is vital for the well-being and survival of evacuees, especially under trying circumstances. This project serves as a model for future humanitarian interventions, emphasizing the importance of addressing overlooked healthcare issues once the immediate crisis has passed, and the need for strategic planning in health care policy for similar emergency scenarios.

## Background

When running for your life, many things may be forgotten or left behind. The question: is where do you rank your concerns about your vision care, and how do you cope if you are fortunate enough to survive? Furthermore, what provisions need to be made for surviving evacuee and refugee populations?

On Oct. 7th, 2023, Hamas attacked multiple Israeli towns on the border with Gaza, killing over 1,200 people, wounding thousands and taking at least 240 hostages [1]. As a result of this incursion into Israel, 70,000 Israelis were forced flee their homes in Southern Israel for safer regions of the country [2]. The remote Dead Sea area had to immediately host ~ 15,000 of these evacuees in hotels, youth hostels and kibbutzim. This area has the advantage of being out of range of Hamas missiles, thus providing the evacuees with relative peace. However, the area is sparsely populated with very little health care infrastructure. Israeli health maintenance organizations (HMO) set up several primary care services in the area, but this did not include many secondary care specialisties such as ophthalmology. The National Health Care System does not cover glasses.

The evacuees escaped without being able to bring personal items. Many of their homes were burned to the ground and/or pillaged. Most cannot return due to the danger of Hamas attacks and missiles. They were expected to be in the evacuee shelters for three months to a year. Many of the evacuees were in a traumatic state, had family members and friends murdered and/or kidnapped and had survived by hiding in safe rooms while terrorists were attacking their towns.

Unlike evacuees, who may be displaced rapidly, refugees often have a slightly longer period to gather personal belongings. Nevertheless, they encounter persistent challenges in accessing consistent eye care due to ongoing instability and scarce resources in refugee camps.

## Methods

Against this background, many of those that fled did not have their eyeglasses or ocular medicine. They were faced with being in a strange place with poor vision and/or risks of irreversible visual deterioration. To address this serious problem, a consortium of volunteers was formed by the Dept. of Optometry at Hadassah Academic College, the Dept. of Ophthalmology at Hadassah Medical Centre, Shamir Optical Industries and Skymed Medical Equipment. The consortium set up ophthalmic clinics at four different Dead Sea venues, consisting of an interdisciplinary team of 7–10 optometrists, one ophthalmologist, at least one orthoptist and 5–10 technicians and logistic volunteers. The vision exams were carried out on four separate days. The consortium dedicated an entire day to each venue and received patients for approximately 10 h. These days were not consecutive so the volunteers drove back and forth to the Dead Sea area for each clinic day.

These clinics provided full service including comprehensive refractive and vision examination, a full ophthalmological examination, some ocular imaging and free glasses. The clinics were set up in social halls and included the following stations: autorefraction (two instruments), tonometry (puff tonometry or iCare), five visual acuity stations each having a portable electronic visual acuity chart (4 m), trial frame and lens case, retinoscope and other small optometric equipment, one slit lamp and one binocular indirect ophthalmoscope. In addition, hundreds of glasses frames were brought and presented for the evacuees for choosing their glasses, and also daily and monthly contact lenses in various base curves.

The social coordinators at each evacuee center sent message to the evacuees under their care inviting them to participate in the vision exam service. Therefore, presentation was based on the patients’ perception of their visual health and needs.

Patients registered for an intake examination where a short ocular and medical history was taken to triage their care. Patients who needed to see an ophthalmologist were flagged at this point. All patients underwent autorefraction (performed by a technician or optometrist) and everyone above age 40 was tested for Intra-Ocular Pressure (IOP) by an optometrist. Subjective refraction and near and distance visual acuity was provided by optometrists who also took a more comprehensive medical history to triage patients to a particular ophthalmologist. Young children were examined by the orthoptist.

Each patient was discharged with a summary for their medical records. This was not a research study so the results were not systematically collected or collated by the researchers.

## Results

Altogether this consortium examined ~ 800 evacuees and provided ~ 700 pairs of glasses. In addition, four patients were referred to ophthalmic emergency departments where they received vision saving treatments due to suspected acute angle closure glaucoma, high IOP, vitreous hemorrhage in a diabetic patient and a suspect retinal detachment. Approximately 40 evacuees received urgent referrals for further investigation at tertiary ophthalmic clinics with most being referred for cataract (*N* = 13), retinal issues (*N* = 10), anterior segment disorders (*N* = 8), and glaucoma (*N* = 5). Pharmaceuticals were provided to patients who were missing their medicines.

The project involved many cases that highlight the psychological and social aspects of vision. For example, a 7-year-old girl whose father, a policeman, was murdered on October 7, subsequently reported visual impairment. The results of the cycloplegic examination showed that the girl had normal visual acuity and refractive error and did not require corrective lenses. However, the girl expressed a strong desire for glasses and cried inconsolably when informed otherwise. The optometrists inferred that the glasses represented a coping mechanism for the girl to deal with the trauma of losing her father. Therefore, she was provided with non-prescription glasses donated by Shamir Optical Industries.

Another example, was a Filipino caregiver who shielded her 95-year-old Jewish patient from harm during a terrorist attack in Kibbutz Nirim. The caregiver lay on top of the patient for several hours to prevent physical and emotional damage. When the terrorists approached them, the caregiver bargained with them and gave them all her money in exchange for sparing the patient’s life. Both survived, but the terrorists smashed the caregiver’s glasses in the process. The project team learned about this incident and sent two optometrists to a nursing home in Jerusalem where the caregiver and the patient were relocated. They examined the caregiver and provided her with two pairs of glasses free of charge within two days.

## Discussion

The evacuees and health care providers considered the eye consortium clinics successful. However, there were several technical challenges. The flow of patients was a particular challenge. The consortium implemented a system in which patients signed up for the exams and were given serial numbers, which were called out by volunteers. This improved patient flow but still resulted in long waiting time for many patients. The issue of patient flow should be considered in future missions. The visual acuity/subjective refractions stations presented a bottleneck for patient flow. There were only five stations due to a limited number of volunteers and space at each venue. Furthermore, many of the patients required triage to the ophthalmologist presenting an additional bottleneck. This could have been overcome by bringing more volunteers and an additional slit lamp and binocular indirect ophthalmoscope. Another technical challenge was that the consortium did not have access to the patients’ HMO medical records and could not update these records with the results of the exam.

Since many of the patients needed to talk with the consortium members about their traumatic experiences, each clinic day ended in a meeting where the team could reflect. In retrospect, it would have been helpful to have a social worker present to help the team members process the horrific stories told by the survivors.

One of the lessons learned from the COVID 19 pandemic, when people avoided medical intervention, is that it leads to irreversible vision problems [3]. The consortium hopes to have mitigated that threat for the evacuees and can serve as a paradigm for further humanitarian interventions.

However, this voluntary effort had both advantages and disadvantages. The advantages of a team of volunteers is flexibility and the ability to provide care immediately without bureaucracy. An inherent disadvantage, however, is that the results of the examination were not entered directly into the patients’ HMO electronic medical record, although patients were provided with a written record. This limited the team’s ability to provide follow-up and monitor patients. A further disadvantage is that it is not sustainable – all consortium members donated their time and Shamir Optics donated the ophthalmic lenses. This is particularly challenging since Shamir Optics is located on the Northern Border of Israel where an additional 60,000 evacuees have had to flee their homes [2], limiting Shamir’s access to their factory. Finally, evacuating people to a remote area increased their personal safety, but reduced their access to general medical care, in contrast to that available for evacuees relocated to large cities, such as Jerusalem and Tel Aviv.

*Policy implications and recommendations for health policy decision makers*:

1. This report highlights the importance of **planning and preparing** for potential eye care emergencies, such as conducting risk assessments, developing contingency plans, allocating funds and resources, and establishing partnerships and collaborations among different stakeholders. This will lead to the establishment of a **national emergency vision care network** that can be activated in times of crisis and coordinate the deployment of qualified eye care professionals and equipment to the affected areas. This network should have a system for **monitoring and evaluating** the eye care interventions, such as collecting and analyzing data on the eye health status, needs, and outcomes of the evacuees, as well as the process, quality, and impact of the eye care services. The data should be linked to the patient’s HMO electronic health records.
2. The evacuees from the recent conflict faced multiple risk factors for developing ocular problems, such as exposure to dust, smoke, and debris, lack of hygiene and sanitation, stress and anxiety, and disruption of their regular eye care routines. Thus, the health policy decision makers must develop **standardized protocols and guidelines** for the assessment and treatment of common ocular conditions among evacuees, such as dry eye, conjunctivitis, and trauma.
3. Providing a** dequate training and support** for the local health care workers and volunteers who assist the eye care team, including basic eye examination skills, infection control measures, and referral pathways. The eye care team should have regular debriefing and supervision sessions to cope with the stress and emotions they encountered during the mission, as well as opportunities for rest and recovery after the mission.
4. Creating **awareness and education campaigns** for the evacuees and the general population regarding the importance of eye health and the availability of eye care services in the evacuation sites.
5. The **resources required** to implement a similar project are volunteer hours, equipment, glasses, space and logistical support. Estimations of the resources should be based on the number of evacuees served.
6. **Extent and duration of the Response**: The response was tailored to meet the acute and diverse needs of the evacuee population, balancing immediate vision care with psychological support. We suggest that the duration of such programs should be flexible, depending on the scale of the crisis and evolving needs of the affected population. Emphasize the importance of continuous assessment to adapt the program duration accordingly.

This study primarily focused on the vision care needs of Israeli evacuees following the October 7th, 2023, attack. However, similar considerations are relevant for other displaced populations, such as refugees. Unlike evacuees who may be displaced rapidly, refugees often have a slightly longer window to gather personal belongings. Despite this, they face ongoing challenges in accessing consistent eye care due to prolonged instability and limited resources in refugee camps. Both groups are susceptible to vision care disruptions that can lead to severe and potentially irreversible visual impairments. Therefore, it is crucial to implement strategic and adaptable vision care interventions for all displaced populations. This includes establishing emergency vision care networks, developing standardized treatment protocols, and ensuring the availability of necessary equipment and medications. By learning from our experience with Israeli evacuees, we can better prepare for and address the vision care needs of displaced populations globally.

In conclusion, the voluntary consortium’s rapid response to the vision care needs of evacuees and refugees from the recent conflict in Southern Israel serves as a valuable example of humanitarian intervention in times of crisis, highlighting the importance of immediate medical attention to prevent irreversible vision problems and offering insights for future endeavors in similar contexts.

## Conclusions

A consortium of volunteers was established to examine evacuees eyes following October 7th attack on Israel. The consortium consisted of an interdisciplinary team of optometrists, ophthalmologists, orthoptists, technicians and logistic volunteers together with collaboration of Optic industry that provided the equipment for eye testing and glasses for evacuees. Our experience and recommendations highlight that vision care, much like hearing and mobility, is not typically prioritized during evacuations but is crucial for the well-being and survival of evacuees, particularly under trying circumstances. This project underscores the importance of addressing these often-overlooked healthcare issues once the immediate crisis has passed. Our work serves as a valuable example of humanitarian intervention in times of crisis, emphasizing the necessity of immediate medical attention to prevent irreversible vision problems and offering insights for future endeavors in similar contexts.

## Data Availability

Not applicable.

## Abbreviations

HMO: Health maintenance organizations
